# Development of genetic tools for the thermophilic filamentous fungus *Thermoascus aurantiacus*

**DOI:** 10.1186/s13068-020-01804-x

**Published:** 2020-10-10

**Authors:** Raphael Gabriel, Julia Prinz, Marina Jecmenica, Carlos Romero-Vazquez, Pallas Chou, Simon Harth, Lena Floerl, Laure Curran, Anne Oostlander, Linda Matz, Susanne Fritsche, Jennifer Gorman, Timo Schuerg, André Fleißner, Steven W. Singer

**Affiliations:** 1 Biological Systems and Engineering Division, Lawrence Berkeley National Laboratory, 1 Cyclotron Road, Berkeley, CA 94720 USA; 2 Joint BioEnergy Institute, 5885 Hollis Street, Emeryville, California 94608 United States; 3 Institut für Genetik, Technische Universität Braunschweig, Brunswick, Germany; 4 Department of Applied Genetics and Cell Biology, University of Natural Resources and Life Sciences Vienna (BOKU), Muthgasse 18, 1190 Vienna, Austria; 5 Austrian Centre of Industrial Biotechnology (ACIB), Muthgasse 11, 1190 Vienna, Austria; 6 Department of Biotechnology, University of Natural Resources and Life Sciences (BOKU), Muthgasse 18, 1190 Vienna, Austria; 7 College of Natural Sciences, University of Puerto-Rico, Rio Pedras, 17 Ave. Universidad STE 1701, San Juan, 00925 Puerto Rico USA; 8 American High School, 36300 Fremont Blvd, Fremont, CA 94536 USA; 9 Frankfurt Institute of Molecular Biosciences, Goethe University Frankfurt, 60438 Frankfurt Am Main, Germany; 10 École Polytechnique Fédérale de Lausanne, Lausanne, Vaud 1015 Switzerland; 11 Department of Food Science and Technology, University of Natural Resources and Life Sciences Vienna (BOKU), Muthgasse 18, 1190 Vienna, Austria

**Keywords:** Filamentous fungi, *Thermoascus aurantiacus*, *Agrobacterium tumefaciens*, Genetic transformation, CRISPR/Cas9 system, Sexual crossing, Xylanases, Enzyme production

## Abstract

**Background:**

Fungal enzymes are vital for industrial biotechnology, including the conversion of plant biomass to biofuels and bio-based chemicals. In recent years, there is increasing interest in using enzymes from thermophilic fungi, which often have higher reaction rates and thermal tolerance compared to currently used fungal enzymes. The thermophilic filamentous fungus *Thermoascus aurantiacus* produces large amounts of highly thermostable plant cell wall-degrading enzymes. However, no genetic tools have yet been developed for this fungus, which prevents strain engineering efforts. The goal of this study was to develop strain engineering tools such as a transformation system, a CRISPR/Cas9 gene editing system and a sexual crossing protocol to improve the enzyme production.

**Results:**

Here, we report *Agrobacterium tumefaciens*-mediated transformation (ATMT) of *T. aurantiacus* using the *hph* marker gene, conferring resistance to hygromycin B. The newly developed transformation protocol was optimized and used to integrate an expression cassette of the transcriptional xylanase regulator *xlnR*, which led to up to 500% increased xylanase activity. Furthermore, a CRISPR/Cas9 gene editing system was established in this fungus, and two different gRNAs were tested to delete the *pyrG* orthologue with 10% and 35% deletion efficiency, respectively. Lastly, a sexual crossing protocol was established using a hygromycin B- and a 5-fluoroorotic acid-resistant parent strain. Crossing and isolation of progeny on selective media were completed in a week.

**Conclusion:**

The genetic tools developed for *T. aurantiacus* can now be used individually or in combination to further improve thermostable enzyme production by this fungus.

## Background

Due to the potentially deleterious impacts of climate change, which is mainly caused by the use of fossil resources, great efforts have been made to explore the applicability of lignocellulosic plant biomass as sustainable alternative to fossil fuels. Lignocellulosic biomass is the most abundant organic material on earth, consisting primarily of the sugar polymers cellulose and hemicellulose and the aromatic polymer lignin [1, 2]. These sugar polymers can be deconstructed by enzymes (cellulases and hemicellulases) into simple sugars that can be further converted into biofuels and other bio-based products using metabolically engineered bacterial and fungal hosts, which reduces our dependence on finite fossil resources [3]. The cost-efficient deconstruction of lignocellulose is currently the biggest obstacle preventing biofuels from becoming competitive to fossil fuels.

Filamentous fungi are efficient lignocellulose degraders, possessing an arsenal of secreted enzymes that digest cellulose and hemicellulose [4]. These organisms have evolved an elaborated sensing system to detect the components of lignocellulosic biomass and fine-tune the expression of cellulase and hemicellulase genes [5]. Therefore, filamentous fungi are the most important industrial cellulase producers [6, 7].

Recently, there is increased interest in establishing thermophilic organisms that secrete thermostable enzymes for the conversion of plant biomass to biofuels [8–10]. The thermophilic fungi *Thielavia terrestris* and *Myceliophthora thermophila* produced enzymes that were more active across all temperatures tested and released more sugars from pretreated plant biomass than the enzymes of the mesophiles *Trichoderma reesei* and *Chaetomium globosum* [11]. In a separate study, enzymes from another thermophilic fungus, *Thermoascus aurantiacus*, demonstrated a higher level of sugar release from ionic liquid-pretreated switchgrass than *T. terrestris* enzymes and showed activities comparable to the commercial enzymatic mixture CTec2 [12]. *T. aurantiacus* was found to secrete high amounts of a lytic polysaccharide monooxygenase (LPMO) [13]. This enzyme has been extensively studied due to its ability to oxidize cellulose chains and has recently been purified from its native secretome [10, 14].

*Thermoascus aurantiacus* is a homothallic fungus that grows optimally at 50 °C. Induction experiments indicated that both cellulases and xylanases were induced by controlled feeding with xylose, suggesting that the regulatory systems for enzyme expression in *T. aurantiacus* had similarities to the regulatory system in *Aspergillus niger* [15]. These initial results make *T. aurantiacus* an intriguing host for thermostable enzyme production. Improving enzyme production and investigating regulation of cellulase and xylanase expression in *T. aurantiacus* is limited by the absence of genetic tools for this promising fungus.

Efficient strain engineering requires genetically tractable hosts. Several methods have been established to genetically engineer filamentous fungi such as protoplast transformation, electroporation, biolistics and *Agrobacterium tumefaciens*-mediated transformation (ATMT) [16]. ATMT relies on the ability of the plant pathogen *A. tumefaciens* to inject DNA into plant cells and other eukaryotic cells. In this manner, various genetic modifications have been made in fungal genomes, including applying CRISPR/Cas9-based gene editing systems [17]. The initial development of the CRISPR/Cas9 system for filamentous fungi often involved the deletion of counter-selectable marker genes such as *pyrG* [18] and *amdS* [19], which allows the fungus to grow in the presence of otherwise toxic 5-fluoroorotic acid or fluoroacetamide, respectively. Sexual crossing is another versatile tool, which accelerates strain engineering through combining desired phenotypes, mapping genomic loci, removing undesired mutations and generating genetically uniform fungal homokaryons [20, 21]. Notably, sexual crossing is not possible with a variety of industrially highly relevant fungi, and a sexual cycle was only recently established for the classic cellulase producer *T. reesei*, however, not including the industrial strains such as Rut-C30 [22–24].

Genetic tools have been successfully applied to generate high-enzyme-secreting strains. A *Penicillium oxalicum* strain with strongly increased cellulase production was generated through overexpression of *clrB* and deletion of the cellulase repressors *creA* and *bglR* [25]. This strain displayed equal enzyme production as the industrial cellulase-hypersecreting *P. oxalicum* strain JU-A10-T, which was generated through classical mutagenesis. Increases in cellulase and xylanase secretion were also achieved through overexpression of x*lnR* and *clrB* and deletion of *creA* in this fungus [26]. Similarly, a *M. thermophila* cellulase-hypersecreting strain was recently generated by deleting four genes through CRISPR/Cas9-based editing [19]. These examples show the extraordinary potential of genetic strain engineering strategies based on the knowledge of cellulase gene regulation.

Notably, regulation of enzyme coding genes can vary substantially among related fungal species. The transcriptional activators for cellulolytic genes are encoded by *clrB*  in *A. niger* and *P. oxalicum* and *clr-2* in *Neurospora crassa* [26, 27]. Another  transcriptional regulator is *clrA,* whose deletion in *A. niger* had a minor effect on plant biomass deconstruction compared to the deletion of *clrB*, while deletion of its orthologue *clr-1* in *N. crassa* led to strongly impaired cellulase production and severe growth defects on cellulose and cellobiose [28, 29]. The transcription factor XlnR and its orthologues are involved in regulation of xylanolytic genes in *A. niger, P. oxalicum*, *T. reesei* and *N. crassa* [26, 30, 31]. In *A. niger*, XlnR is also involved in the activation of cellulolytic genes [2, 29]. In *T. reesei*, the *xlnR* homolog *xyr*-*1* is the most important regulator of cellulases and xylanases, and its deletion leads to severe growth defects on cellulose [32]. These results make those genes attractive targets for strain engineering purposes.

Development of genetic tools to improve the regulation of plant cell wall degrading enzymes in filamentous fungi provides a pathway to engineer a wider variety of hypersecreting fungal strains. The goal of this study was to (1) develop genetic tools, namely an ATMT-based transformation system, the CRISPR/Cas9 system and a sexual crossing protocol, for *T. aurantiacus* and (2) employ those tools to manipulate the xylanase regulator *xlnR* for a proof of principle study for strain engineering of xylanase secretion. Here, we report on successful establishment of those objectives: an ATMT procedure was established, which was then used to implement the CRISPR/Cas9 system in *T. aurantiacus* by inactivating the native *pyrG* gene through mutations caused by the Cas9 endonuclease. Lastly, a sexual crossing protocol has been developed for this fungus, allowing rapid combination of genetic modifications within a week. As a proof of concept, we generated high-xylanase-secreting strains via integration of a *xlnR* cassette into the fungal genome with ATMT, displaying the applicability of the developed methods for generating high-enzyme-secreting *T. aurantiacus* strains for cost-efficient biofuel production.

## Results

### *Agrobacterium tumefaciens*-mediated transformation system development for *T. aurantiacus*

Various transformation protocols have been developed for filamentous fungi, such as protoplast generation, electroporation, ATMT and nanoparticle-based methods such as biolistics [16]. Attempts to transform *T. aurantiacus* by protoplastation and electroporation were unsuccessful (data not shown). Therefore, ATMT was chosen for the transformation of *T. aurantiacus.*

**Fig. 1:**
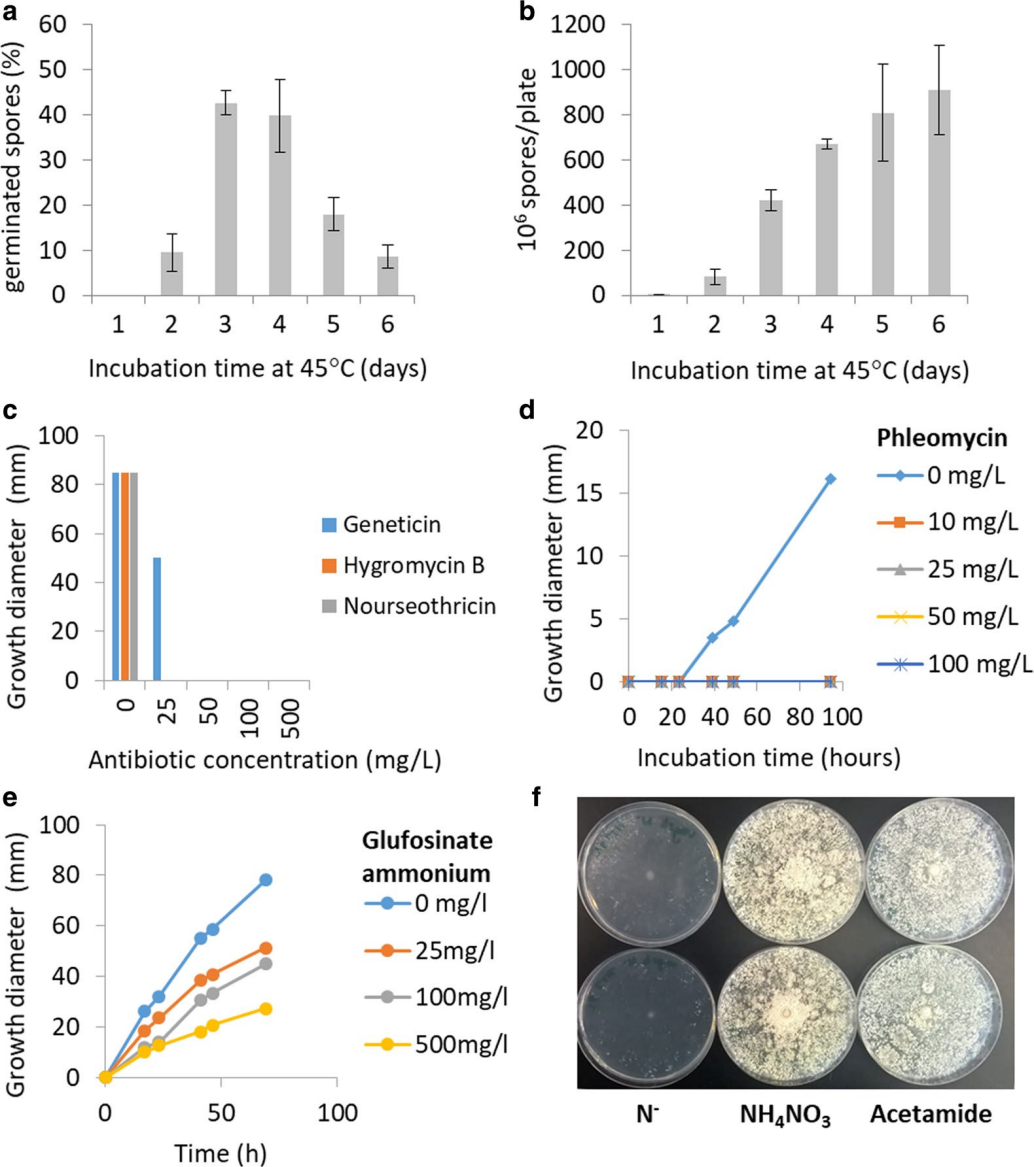
Ascospore production and antibiotic susceptibility of *T. aurantiacus*: **a** Germination rates were assessed from spores of fungal cultures incubated at 50 °C for 2 days and then 45 °C for 1–6 days. At the indicated time, spores were scraped from 3 replicate plates for each day; germination was detected via randomized counts of spore suspensions. **b** The total amount of produced spores was calculated with a hemocytometer. Growth tests of *T. aurantiacus* on different selection markers: **c** hygromycin B, nourseothricin and geneticin (PDA medium), **d** phleomycin (Vogel’s minimal medium) and **e** glufosinate ammonium (Vogel’s minimal medium). **f** *T. aurantiacus* is able to grow on acetamide (Vogel’s minimal medium with no nitrogen added, ammonium nitrate and acetamide from left to right). Two replicate plates were used for all assays. Note that all antibiotics or acetamide were separately sterile filtered and added to the media after autoclaving. Two biological replicates were used for each test

*Thermoascus aurantiacus* is a homothallic fungus and was reported to only produce ascospores for propagation through self-crossing [33]; no conidiospores have been observed for this species. It was found that ascosporogenesis in this fungus was similar to those described for ascomycetes [34]. ATMT involves the co-cultivation of the bacteria with germinating spores of the fungus. We, therefore, first determined optimal culture conditions for *T. aurantiacus* ascospore production by testing the growth media PDA, Vogel’s minimal medium and YPD (data not shown). Spore production was found to be as follows: PDA > Vogel’s minimal medium > YPD. Since PDA yielded the largest number of spores, it was chosen for the following experiments. In the next step, we tested different pre-culture conditions for optimal spore production and germination rates. The most efficient spore germination was found when spores were harvested from PDA plates grown for 2 days at 50 °C and 3 to 4 days at 45 °C (Fig. 1a). However, a higher spore yield was obtained from plates, on which *T. aurantiacus* was grown for 4 days at 45 °C (~ 7 * 10^8^ spores per plate, see Fig. 1b). Therefore, the latter incubation time was chosen to harvest spores for ATMT.

Transformation of fungi usually involves the selection of transformants via antibiotic resistance markers [35]. Commonly used antibiotic resistance genes confer resistance to hygromycin B, nourseothricin, glufosinate ammonium, geneticin and phleomycin [16, 36]. Alternatively, acetamide can be used as a nitrogen source to isolate successful transformants through integration of an acetamidase gene (*amdS*), since not all species possess this essential enzyme for acetamide utilization [37]. To test the potential application of these selection systems for *T. aurantiacus*, the basic resistance level of the wild-type strain against the above-mentioned antibiotics was determined. In addition, we tested if the fungus can grow using acetamide as the sole nitrogen source. Strong growth inhibition was observed on plates containing hygromycin B, nourseothricin, geneticin and phleomycin, while the fungus was able to grow on Vogel’s medium supplied with glufosinate ammonium (Fig. 1c–e). *T. aurantiacus* showed robust growth on minimal media plates supplemented with acetamide as the sole nitrogen source (Fig. 1f), which was consistent with the presence of a putative *amdS* gene (Theau2|635859) in the *T. aurantiacus* genome (https://mycocosm.jgi.doe.gov/Theau2/Theau2.home.html).

**Fig. 2:**
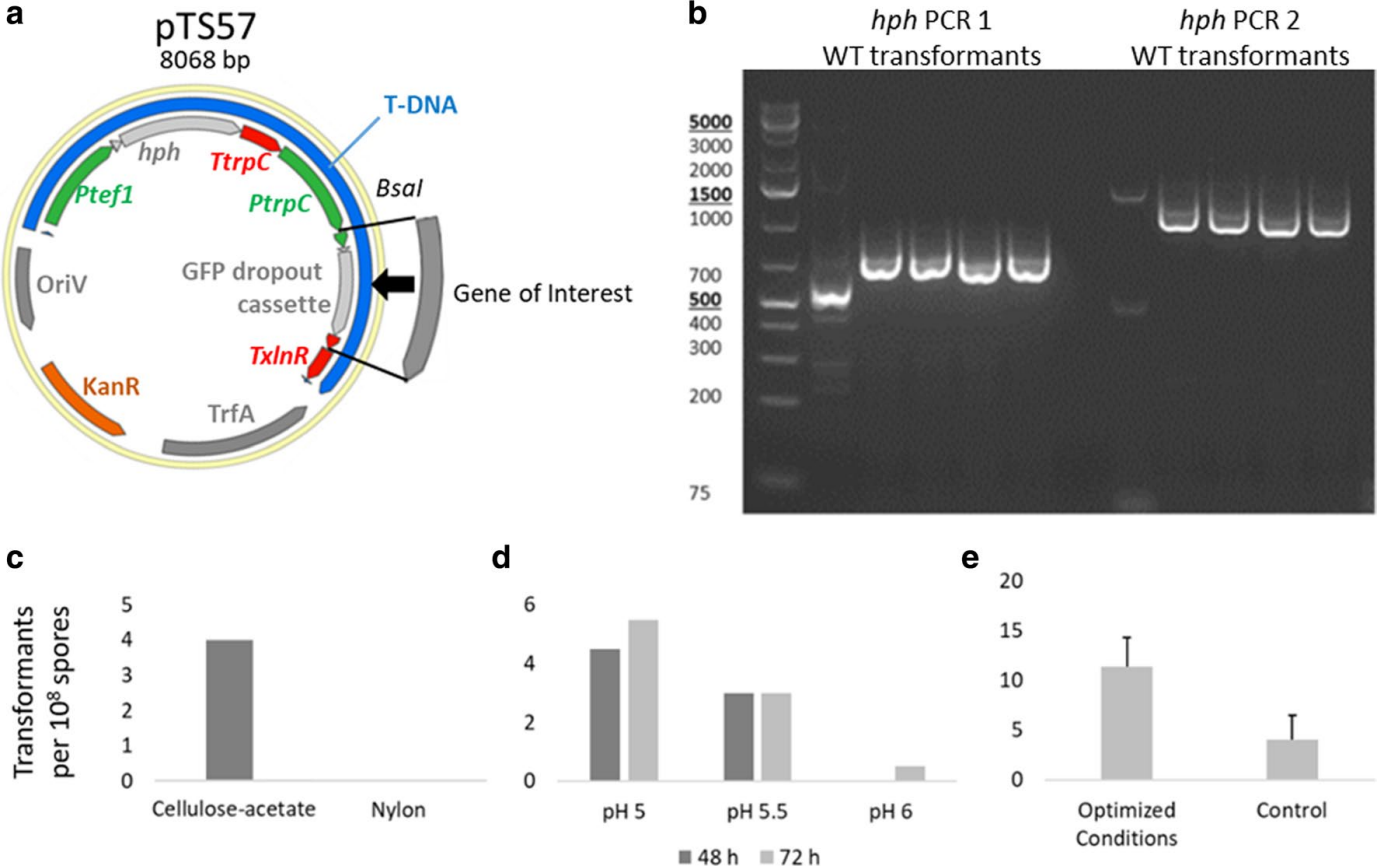
**a** The ATMT plasmid pTS57 was designed for efficient insertion of genes of interest and screening through Golden Gate Cloning upon replacement with a GFP-drop-out cassette. The gene of interest is expressed with the native *T. aurantiacus gpd* promotor and *xlnR* terminator, the *hph* gene is expressed with the native *T. aurantiacus tef*-*1* promotor and *trpC* terminator. **b** PCR analysis to verify the *hph* integration into *T. aurantiacus* via ATMT. Optimization of the ATMT procedure for **c** membrane used, and **d** incubation time and pH. **e** A combination of optimized pH and temperature was tested regarding transformation rates. (c: 1 biological replicate, d: 2 biological replicates, and e: 3 biological replicates). Error bars indicate the standard deviation of 3 biological replicates

For the first approach to establish ATMT, the Golden Gate-compatible plasmid pTS57 (Table 1) was constructed to mediate ectopic integrations of genes of interest into the fungal genome and to allow selection using hygromycin B resistance (Fig. 2a). In the pTS57 plasmid, the *hph* gene is driven by the native *T. aurantiacus tef*-*1* promotor and there is a cloning site for genes of interest expressed by the native *T. aurantiacus gpd* promotor.

**Table 1:** List of plasmids used in this study

Name	Bacterial marker	fungal marker	Parent plasmid	Insert	ICE repository ID
pTS57	Kan	*hph*	NA	*gfp*	JPUB_017131
pTS67	Kan	*hph*	pTS57	*xlnR*	JPUB_017129
pJP1	Kan	*hph*	pTS57, JP36_1	*Cas9, gRNA 1*	JPUB_017147
pJP3	Kan	*hph*	pTS57, JP36_3	*Cas9, gRNA 3*	JPUB_017149

A previously developed ATMT protocol for *Rhodosporidium toruloides* [38] was modified for transformation of *T. aurantiacus* (for details see the “Methods” section). Briefly, 10^8^ fungal spores were mixed with 2 ml of an induced *A. tumefaciens* culture of OD_600_ of 1 carrying the plasmid pTS57 and incubated on a filter for 48 h on induction agar containing acetosyringone. After incubation, the spores were washed off the filters and spread on hygromycin B PDA containing cefotaxime to remove remaining bacteria. The grown fungal colonies were isolated after 2 days of incubation. In the initial experiment, four transformants were obtained from cellulose acetate filters using 10^8^ spores while no transformants were obtained when using a nylon filter (Fig. 2c). The presence of the hygromycin B resistance gene *hph* in all four strains was PCR verified (Table 3) through two different primer sets (Fig. 2b, Table 2). Thus, this initial transformation approach was successful; however, transformation frequency was low.

**Table 2:** List of primers used in this study

PCR	Name	Sequence
*hph1*		
FWD	RG1	CTCGGAGGGCGAAGAATCTC
REV	RG2	ATTTGTGTACGCCCGACAGT
*hph2*		
FWD	TS222	CGTAGTACCTGAGCACCCCTCTGAGCTCTT
REV	TS223	CCATTTGTCTCAACTCCGGAGCTGACATCGA
*pyrE*		
FWD	RG75	GACGGTTTCTATACAGTCTTTTCAG
REV	RG76	CCCCCGATGTTACTCCGC
*pyrG*		
FWD	LLK683	TTCTTACTACAACTTGGCAACCTTC
REV	LLK686	ACAAGCCAAATTACCAGCAGAATAC

The influence of the pH of the induction medium, the time of co-cultivation of the fungus and the bacteria, and the cultivation temperature were tested to further optimize the transformation protocol. We found that reducing the pH of the induction medium from 5.5 to 5 yielded on average 1–2 more transformants per 10^8^ spores, while varying the incubation time (48 vs 72 h) had virtually no effect on the number of transformants obtained (Fig. 2d). The temperature test indicated that increasing the temperature from 26 °C to 28 °C led to slightly higher transformation rates (data not shown). The combination of changing the induction medium pH to 5 and raising the incubation temperature to 28 °C led to the isolation of ~ 2.5 times more colonies compared to the initial conditions of pH 5.5 and 26 °C (Fig. 2e).

### Genomic integration of *xlnR* expression cassettes lead to increased xylanase secretion

**Fig. 3:**
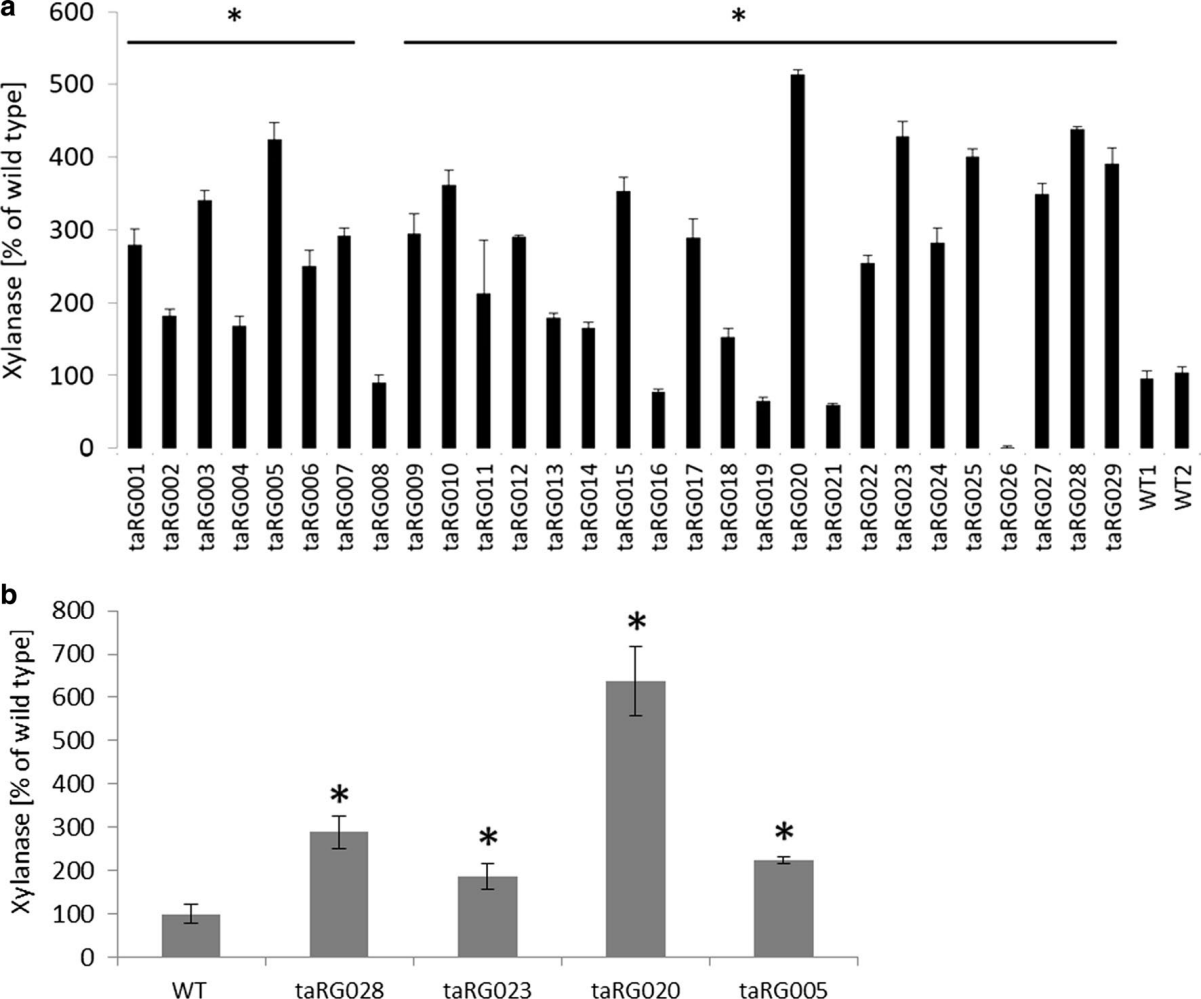
Xylanase activity of the *T. aurantiacus* strains transformed with a *Pgpd*::*xlnR* construct. **a** The DNS Assay was used to screen 29 transformants grown in Avicel medium for xylanase. **b** From a subset of the mutants tested in (**a**), a subset of 4 mutants displaying the highest xylanase activity was used for a shift experiment. The mutants were grown in McClendon’s medium supplemented with soy meal peptone and glucose for 48 h and equal amounts of mycelium were shifted to starvation medium for 72 h and xylanase activity was measured. **a** Bars represent one biological replicate and the error bars are the standard deviation of 3 technical replicates, the horizontal bars with the asterisk indicate statistical significant difference to the wild-type strains (*p* value < 0.05), **b** bars and standard deviation are derived from three biological replicates, the asterisk indicate statistical significant difference to the wild-type strains (*p* value < 0.05)

After establishing the ATMT procedure of *T. aurantiacus* ascospores, we used the method to demonstrate a proof of concept approach for the expression of a gene of interest in *T. aurantiacus*. Previous work had demonstrated that a continuous xylose feed induced both cellulase and xylanase activities in *T. aurantiacus*, raising the question of the involved transcriptional regulators [15]. In *T. reesei*, the transcription factor Xyr1 acts as an activator for xylanases and cellulases. This regulatory function is conserved for the respective homologs in different ascomycete species [32, 39, 40]. A *xyr1* homolog (Theau2|210222), named *xlnR,* had been identified in the *T. aurantiacus* genome in an earlier study [10]. To test the function of this regulator, we cloned the *xlnR* open reading frame into pTS67, where the gene is expressed by the native *T. aurantiacus gpd* promotor (Table 1). The plasmid was transformed into the wild-type reference strain using the established ATMT protocol. 29 hygromycin B-resistant transformants were obtained. For a subset of 16 isolates, the presence of the resistance gene within the genome was verified by PCR analysis using an *hph*-specific primer pair (data not shown). To test the effect of the newly integrated construct on xylanase activity, the 29 transformants and the wild-type recipient strain were cultured in liquid media containing Avicel cellulose, a substrate that poorly induces xylanase, as the sole carbon source and xylanase activity was determined after 3 days of cultivation. For 24 out of the 29 isolates, a > 50% increase in xylanase activity was observed, and 10 transformants out of this group demonstrated a > 300% increase with one transformant displaying a 500% increase (Fig. 3a). The secretion of elevated amounts of xylanase by the transformants was also tested under non-inducing conditions. A shift experiment was performed with 4 isolates that displayed the highest amount of xylanase activity during incubations on Avicel celluose. These strains and the wild type were grown in glucose medium first and equal amounts of fungal biomass were then shifted to carbohydrate-free medium. We found a sixfold increase in xylanase activity compared to wild type in these isolates (Fig. 3b). This proof of concept test indicated that enzyme secretion of this fungus could be successfully manipulated with the established ATMT procedure.

### Development of a sexual crossing protocol for *T. aurantiacus*

**Fig. 4:**
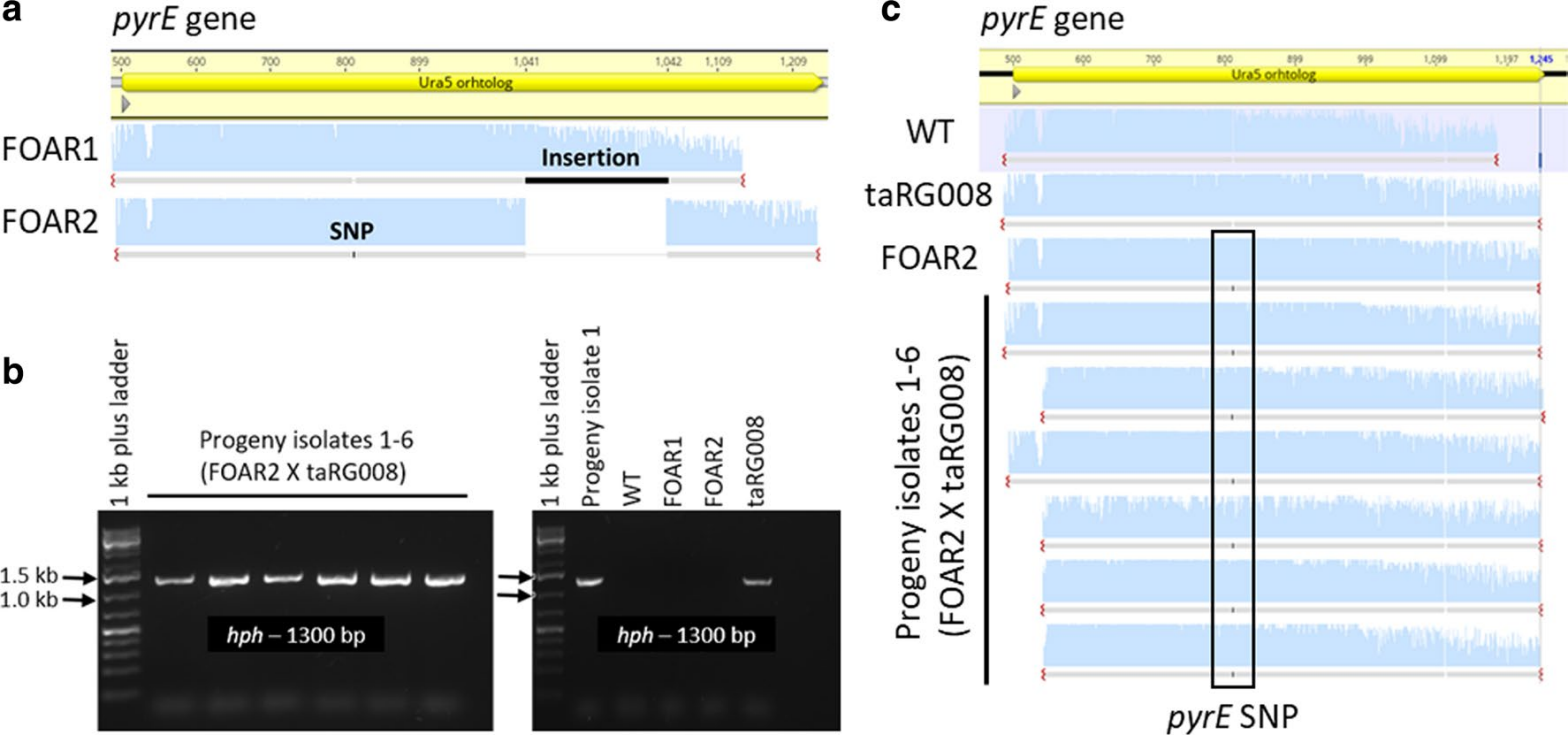
Testing sexual outcrossing in *T. aurantiacus*: **a** Sequencing data of the *pyrE* gene of two 5-FOA-resistant isolates (FOAR1-2) were aligned to the *pyrE* reference sequence (primers are listed in Table 2). FOAR2 was crossed with the hygromycin B-resistant strain taRG008 (*hph* strain). The progeny of this cross was analyzed through **b** PCR amplification of the *hph* gene (wild type, FOAR strains and taRG008 were included as controls). **c** Sequencing data of the *pyrE* gene sequence of the crossed strains in (**b**) were aligned to the native *pyrE* reference sequence. The sequence analysis was performed with Geneious version 11.1 (Biomatters). This analysis indicated that only the progeny isolates displayed genomic integrations of the *hph* gene and the *pyrE* mutation from FOAR2

*Thermoascus aurantiacus* is a self-fertile, homothallic fungus, which completes its sexual life cycle without a crossing partner. However, other homothallic species, such as the model fungus *Sordaria macrospora*, are often able to outcross [41]. In these cases, the basis for outcrossing is the formation of heterokaryotic mycelia via vegetative hyphal fusion of genetically compatible strains. Completion of the sexual cycle of these heterokaryons gives rise to genetically recombinant progeny. To test if outcrossing occurs in *T. aurantiacus* and to establish a crossing protocol, two strains with different selectable markers were employed. The hygromycin B-resistant *T. aurantiacus* strain taRG008 (Fig. 3a) carrying the *xlnR* expression cassette described above was chosen as one of the crossing partners. For the second crossing partner, UV mutagenesis of *T. aurantiacus* ascospores was performed to isolate mutants that were uracil auxotrophs and resistant to 5-fluoroorotic acid (5-FOA). Metabolism of 5-FOA by wild-type fungi generates the toxic intermediate fluorodeoxyuridine. 5-FOA, therefore, selects for mutants with non-functional *pyrG*, which encodes for orotidine 5′-phosphate decarboxylase and *pyrE*, which encodes for orotate phosphoribosyltransferase [42, 43]. The orthologues of *pyrE* and and *pyrG* in *T. aurantiacus* are Theau2|404792 and Theau2|629805, respectively. UV mutagenesis yielded two 5-FOA-resistant strains (FOAR1 and FOAR2) that were isolated on 5-FOA minimal medium plates containing uracil. Subsequent sequencing of the *pyrE* gene region identified causative mutations for 5-FOA resistance (Fig. 4a). An insertion of 190 bp was found in FOAR1, which turned out to be a duplication of part of the *pyrE* gene sequence, while FOAR2 had a 1 bp insertion in *pyrE*, which created a frameshift mutation for both strains. FOAR2 was chosen as the partner to be crossed with the hygromycin B-resistant strain taRG008 (Fig. 3 a). Recombinant progeny were expected to harbour both resistances that could be easily screened for on media supplemented with hygromycin B, uracil and 5-FOA. The plate set-up for fungal crossings is shown in Additional file 1: Fig. S1. Briefly, 2 fungal strains were inoculated on a PDA plate supplemented with uracil in alternating fashion to maximize the possibility to form a contact interface. From this interface that was expected to contain the crossed spores of both strains, the mycelium was scraped off the surface with a spatula and eluted in water. The spores were released through vortexing and filtered. Different dilutions were made and spread onto squared agar plates containing hygromycin B, 5-FOA and uracil to yield very few (< 10) growing colonies, which simplified the isolation. Six progeny colonies (P1–6) were randomly isolated for further analysis on the selective plates. Genomic DNA was extracted from those colonies and was used to verify the integration of the *hph* gene cassette that was passed on from parent strain taRG008 (Fig. 4b, left gel) as well as the *pyrE* mutation of the parent strain FOAR2 (Fig. 4c). The wild type, FOAR1, FOAR2, the *xlnR*/*hph*-expressing strain taRG008 and one progeny isolate (P1) were included as controls for both PCRs (Fig. 4b, right gel). The PCR amplification of the *hph* gene and Sanger sequencing of the *pyrE* PCR confirmed that both modifications were only present in the progeny isolates (Fig. 4c).

### Development of a CRISPR/Cas9 protocol for gene deletion in *T. aurantiacus*

CRIPSR/Cas9 is a powerful genome editing tool consisting of an RNA-guided endonuclease (Cas9) and one or multiple guide RNAs (gRNAs) for targeting one or several genomic loci at the same time [44]. Cas9 can be introduced into the fungal cell in the form of DNA, RNA or a protein–RNA complex. Unlike other commonly used transformation strategies, ATMT only allows transformation of DNA fragments into the fungal cell. Therefore, ATMT-mediated Cas9 introduction into fungal cells relies on genomic integration of the Cas9 and gRNA expression cassettes [45, 46]. To apply Cas9-based editing in *T. aurantiacus*, an AMA1 plasmid-based expression approach from Nødvig [47] was chosen and modified for ATMT. In the present study, Cas9 and gRNA expression cassettes were amplified from AMA1-based Cas9 plasmids generated in the study mentioned before and integrated into the ATMT plasmid pTS57, generating a new series of plasmids (pJP1, pJP3, Table 1). These ATMT-compatible Cas9 plasmids were then used to integrate an expression cassette of the Cas9 gene, the gRNA and the *hph* marker into the fungal genome.

**Fig. 5:**
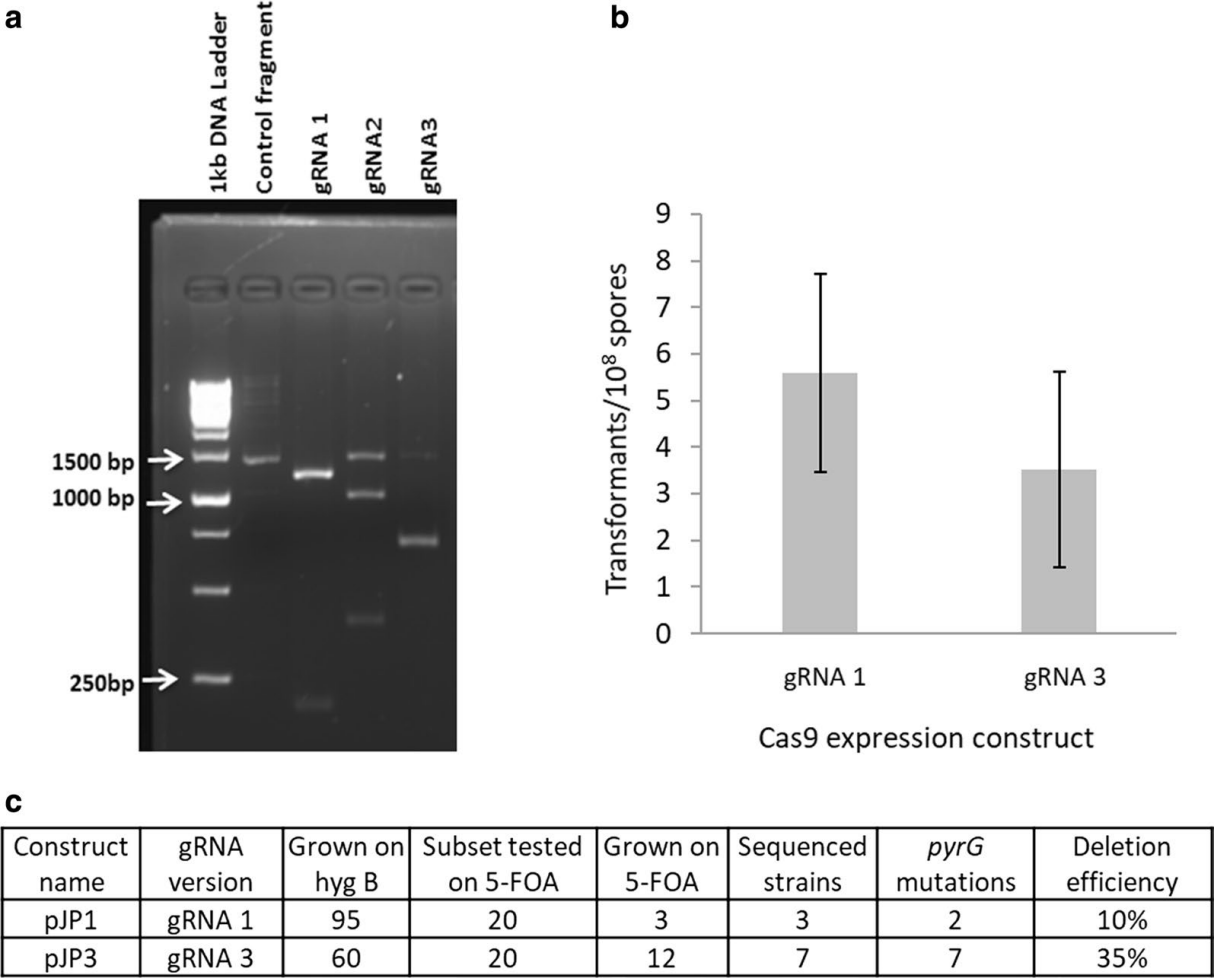
CRISPR/Cas9 development in *T. aurantiacus*: **a** in vitro Cas9 cleavage assay: Agarose gel depicting the uncleaved control fragment and the Cas9 cleavage of the target *pyrG* sequence with gRNA 1, 2 and 3. **b** Transformation efficiency per 10^8^ spores with gRNA 1 and 3 containing vectors, selected on hygromycin B uracil plates. Each bar displays the mean and standard deviations from 17 biological replicates. **c** Deletion efficiencies of both gRNAs targeting Cas9 to the *pyrG* gene in *T. aurantiacus*

To demonstrate the CRISPR/Cas9 gene editing approach in *T. aurantiacus*, the *pyrG* gene was chosen as a target for gene inactivation. Disruption of *pyrG* not only causes uracil auxotrophy but also confers resistance to 5-fluoroorotic acid (5-FOA), which allows the screening of *pyrG* mutants. Three different gRNAs targeting the *pyrG* gene in *T. aurantiacus* were designed using CRISPOR web-tool [39] (Table 3) and first tested in vitro through performing a Cas9 cleavage assay. Accordingly, purified Cas9 protein, a *pyrG* PCR product and one in vitro-transcribed gRNA per reaction were incubated to facilitate *pyrG*-DNA cleavage mediated by the Cas9 ribonucleoprotein complex. Each reaction was then analyzed through agarose gel electrophoresis to visualize the cleaved DNA fragments. The cleavage efficiency was found to be as follows: gRNA 1 > gRNA 3 > gRNA 2 (Fig. 5a). Therefore, gRNA 1, gRNA 3 and the Cas9 gene were cloned into the ATMT plasmid pTS57, yielding the plasmids pJP1 and pJP3, respectively. Transformations of *T. aurantiacus* ascospores using those plasmids were performed by ATMT. The selection for positive transformants was performed through screening for hygromycin B resistance (Fig. 5b) and, on average, approximately 5.5 (pJP1) and 3.5 (pJP3) transformants per 10^8^ spores were obtained. A subset of 20 of each of these transformants were randomly picked and further screened for 5-FOA resistance on Vogel’s MM supplemented with uracil and 5-FOA. Three (pJP1) and 12 (pJP3) out of 20 transformed *T. aurantiacus* isolates displayed 5-FOA resistance (Additional file 1: Figs. S2a, S5c). Sequencing of a subset of isolates from transformations with pJP1 and pJP3 revealed mutations within the protospacer targeting sequence of the *pyrG* gene, confirming that Cas9 cleavage led to base deletions and mismatches next to the PAM sequence, which caused a frameshift in the *pyrG* gene in all sequenced strains and thus 5-FOA resistance of the respective strains (Additional file 1: Fig. S2b). The deletion efficiency was then calculated based on the fraction of *T. aurantiacus* transformants isolated on hygromycin B medium after the ATMT transformation that also had a mutation in the *pyrG* gene: gRNA 1 displayed a deletion efficiency of 10% while gRNA 3 displayed a deletion efficiency of 35% (Fig. 5c). Thus, the Cas9 system successfully introduced mutations in the *pyrG* gene, and selection on 5-FOA turned out to be effective to screen for those mutants.

**Table 3:** List of protospacers and PAM sequences used in this study

Target locus	ID	Protospacer sequence (5′–3′)	PAM (5′–3′)
*pyrG*	gRNA 1	CTTTTGCGCGCGAGCGCCGT	AGG
*pyrG*	gRNA 2	GAGTCTTCCTGCACAGGCCT	GGG
*pyrG*	gRNA 3	TCGGCGCCCGACTTCCCCTA	CGG

## Discussion

In this study, we have established a variety of genetic tools to engineer *T. aurantiacus*. These tools include an ATMT method for transformation, a sexual crossing protocol and a Cas9-based method for gene editing. While genetic tools have been developed for a number of mesophilic filamentous fungi, there are limited genetic tools for thermophilic fungi, so development of genetic tools for *T. aurantiacus* represents the first step towards establishing this fungus as a production platform for thermostable enzymes.

ATMT was a successful approach to transform *T. aurantiacus*; however, the process is more time-consuming than other frequently used transformation approaches and limits the extent of engineering possibilities. Developing protoplast transformation or electroporation protocols for *T. aurantiacus* will accelerate and expand engineering. ATMT was previously established for conidiospores of the thermophilic fungus *M. thermophila* [18] and generated up to 145 transformants per 10^5^ spores. Thus, the transformation rates reached for *T. aurantiacus* ascospores in this study (10 per 10^8^ spores) were significantly lower. The ATMT procedure was then used to genomically integrate an expression cassette of the transcriptional regulator *xlnR*. Transformants carrying the *xlnR* construct exhibited high variability of xylanase activity in the culture supernatants, which would be consistent with random integrations of the cassettes in unknown genomic regions and variable numbers of genetic copies inserted into the genome. Nevertheless, up to 500% increased xylanase activity was observed compared to the wild type in strains carrying the *xlnR* construct. Therefore, the *T. aurantiacus xlnR* appears to have a comparable function to its homologs in the closely related *Aspergillus* spp. and *P. oxalicum*, which regulate xylanase gene expression [26, 29, 40]. Notably, cellulases and xylanases secretion of *A. niger* in the presence of d-xylose was linked to phosphorylation of XlnR, which mediates the induction of the respective genes in the presence of this carbon source [2, 31]. *T. aurantiacus* is closely related to *A. niger* and was found to produce high amounts of cellulases and xylanases during D-xylose fed-batch conditions, which might be mediated by XlnR as well [15].

Furthermore, the ATMT method enabled the successful establishment of the CRIPSR/Cas9 system in *T. aurantiacus*. ATMT is a time-consuming method compared to PEG-mediated protoplastation and electroporation, while no special equipment is needed as in the case of biolistics [35]. Also, ATMT has a higher risk of contamination due to several transfer steps of the filters harboring the bacteria and spores. At the same time, this procedure has enabled the transformation of many fungal species so far and often is the method that enables to access difficult to transform species [48]. To our knowledge, no successful transformation protocols were previously reported for *T. aurantiacus*. ATMT only allows DNA-based transformations, while protoplastation and electroporation can also be used to transform the cell through using proteins and RNA (e.g., transformation of pre-mixed Cas9–gRNA complexes). Nevertheless, ATMT now allows modification of the *T. aurantiacus* genome, which opens up a wide variety of applications of industrial and academic interest. Ultimately, the establishment of more time-efficient strain engineering procedures than ATMT will be of interest. However, even then ATMT can still be of high utility due to the possibility to perform functional genomics through random T-DNA integration and subsequent strain screening and verification of the integration sites can then be used to uncover new target genes [48].

The gene editing system relied on the non-homologous end joining (NHEJ) repair pathway to generate mutations in the *pyrG* gene, which has been previously demonstrated in *M. thermophila* for the *amdS* gene [19]. Additionally, the ability to generate protoplasts for *M. thermophila* led to the introduction of multiple plasmids, which permitted deletions of genes using homology-directed repair (HDR) mechanisms with a *ku70* deletion strain [18]. Nonetheless, the CRISPR/Cas9 system can now be used to modify and investigate the role of other well-known regulators, such as *creA*, *clrA*, *clbR* and *amyR* in a multiplexed manner to further uncover cellulase and xylanase regulation in *T. aurantiacus* [29, 36, 40, 49, 50]. Moreover, other genes related to carbon catabolite repression and secretion of other carbohydrate active enzymes might be vital targets for understanding and engineering CAZyme secretion in *T. aurantiacus* [51, 52]. Such genes can be found through systems biology methods such as RNA-Seq, which will enable the identification of novel CAZyme regulators in *T. aurantiacus* [53]. Finally, recyclable markers such as *pyrG* allow to delete target genes with a high efficiency and then remove the marker through a loop-out mechanism by adding homology repeats [54].

The demonstration of sexual crossing between two strains of *T. aurantiacus* reveals an important advantage for this fungus as a potential platform for producing thermostable enzymes. Crossed progeny were readily isolated on double selection media to isolate strains that possess both an *hph* cassette and a mutated *pyrE* gene. Sequencing results revealed that all isolated strains carried the parent-specific *pyrE* frameshift mutation together with *hph*. In conclusion, our results are the expected outcome of a successful sexual cross. One shortcoming of the chosen approach was that the location of the *hph* cassette was unknown. Thus, the potential linkage between *hph* and *pyrE* could have affected the efficiency of the crossing. Nevertheless, the main goal here was to obtain a proof of concept for combing *hph* and the mutated *pyrE* into one strain. For strain construction using this protocol, assessing the location of both selection marker genes can be of further utility.

Crossing under laboratory conditions is a valuable genetic tool only available for a limited number of species, such as the model fungi *N. crassa* [55], *A. nidulans* [56] and very recently also *T. reesei* [23], but is lacking for several industrially relevant fungi with unknown teleomorphs such as *A. niger* and *A. oryzae* [22, 24]. *M. thermophila* is not capable of self-crossing and does not cross with close relative *Myceliophthora heterothallica*, which has been experimentally demonstrated to have a sexual life cycle [57, 58]. An additional advantage of the homothallic *T. aurantiacus* is that crossing does not require strains with different mating types as in heterothallic fungi like *T. reesei* [23] or *P. chrysogenum* [59]. Ascospores are the only means of propagation in *T. aurantiacus* and are produced in as little as 4–5 days. Since ascospores originate from a single nucleus, the resulting progeny are always homokaryotic, allowing for quick and simple purification of originally heterokaryotic transformants. Additionally, the sexual crossing of *T. aurantiacus* was demonstrated on conventional fungal media such as PDA. Since crossed transformants were isolated within a week, it appears that the crossing procedure with this fungus is substantially faster and easier than procedures used for other fungi such as *N. crassa* or *A. nidulans* [60].

In summary, the developments demonstrated in this paper will enable rapid stacking of genetic modifications into new strains for subsequent strain tests. We expect these developments and further improvements of the genetic transformation procedure to turn *T. aurantiacus* into a novel host for studying plant cell wall deconstruction, sexual biology and cell biology. In addition, these protocols provide the basis for developing *T. aurantiacus* as a host for numerous biotechnological applications.

## Conclusion

The methods generated in this study will enable to substantially expand the use of *T. aurantiacus* in both applied and fundamental studies. *T. aurantiacus* is an intriguing host for cellulase production due to the extraordinary thermostability of its cellulases, the high enzyme titers secreted by the wild type and, since it is a homothallic fungus, the possibility to rapidly cross strains carrying different mutations into homokaryotic progeny in substantially shorter time frames than currently used industrial fungi, thereby enhancing strain engineering. With further development regarding the transformation system, CRISPR/Cas9, and the crossing protocol, it will be possible to generate genetically modified strains that can be crossed to combine desired mutations. This will enable high CAZyme production with *T. aurantiacus* through deleting or overexpressing regulators and other genes known to impact CAZyme production in related filamentous fungi.

## Methods

### Chemicals

All chemicals were purchased from Sigma-Aldrich unless otherwise indicated.

### Strains and culture conditions

*Thermoascus aurantiacus* ATCC^®^ 26904™ was obtained from the American Type Culture Collection and grown on TEKNOVA potato dextrose agar (PDA) plates to obtain ascospores for transformation purposes. The PDA plates were inoculated with ascospores and incubated for 2 days at 50 °C before they were transferred to 45 °C for another four days. This shift was performed due to elevated evaporation of PDA plates at 50 °C. The plates were covered with a glass beaker to reduce drying, and plastic containers filled with distilled H_2_O provided a moist atmosphere. Cultivation of the uracil auxotroph strains generated in this study was performed on solid Vogel’s minimal medium containing Vogel’s salts solution, 2% sucrose and 1.5% bacto agar supplemented with 1 g/L uracil and 1 g/L 5-FOA as indicated.

*Agrobacterium tumefaciens* strain EHA105 was grown in Luria–Bertani (LB) medium plates (supplemented with kanamycin at 50 µg ml^−1^ when culturing transformed strains harboring plasmids for the fungal transformations). After 2 days, 2–3 *A. tumefaciens* colonies carrying the desired plasmids were inoculated in 10 ml of liquid LB medium at 30 °C supplemented with kanamycin as described above.

### Antibiotic resistance plate tests of *T. aurantiacus*

For all plate tests, counted *T. aurantiacus* spores were placed in the center of 9 mm agar plates containing the desired antibiotic. These plates were then incubated at 45 °C, and fungal growth was measured after 72 h (hygromycin B, geneticin, nourseothricin, 5-fluoroorotic acid [5-FOA] and 5-fluoroacetamide [5-FAA]) or as indicated (glufosinate ammonium and phleomycin). The fungal mycelium diameter was measured with a vernier caliper from two sides and averaged. Antibiotic concentrations were added as indicated or 1.3 mg/ml for 5-FOA and 5-FAA. All antibiotics were sterile filtered separately and added after sufficient cooling of the agar. The following media compositions were used: PDA for hygromycin B, geneticin and nourseothricin, Vogel’s minimal medium with 2% sucrose for glufosinate ammonium, phleomycin (pH 8), 5-FOA and 5-FAA.

### Ascospore production and germination rate tests

PDA plates (Sigma-1879 V) were inoculated in 3 biological replicates for each time point and ascospores were incubated at 50 °C for 2 days and 1–6 days at 45 °C. Spores were harvested through scraping off the surface with a cell spreader two times and filtering through miracloth. These spores were counted with a hemocytometer, diluted appropriately, spread on a new plate and incubated for 16 h at 45 °C. These spores were then randomly imaged with a Leica-DM4000B microscope, and the germination rate was calculated via counting germinated versus non germinated spores with ImageJ [61]. A minimum of 385 spores was counted for each day, except for day 3, where almost no spores were present.

### Plasmid design and cloning strategy

The base vector pTS57 (P*gpd*::P::*gfp*::T::T*xlnR*; P*tef1*::*hph*::T*trpC*) was used to generate all further vectors (see Table 1). pTS57 expresses the gene of interest with the *T. aurantiacus gpd* promotor and *xlnR* terminator, which flank a *gfp*-dropout cassette that is recognized by *E. coli*. This cassette has two *BsaI* restriction sites at either end and allows genes of interest to be inserted through Golden Gate Cloning. *E. coli* transformants harboring the plasmid with the integrated gene of interest can then be identified through loss of *gfp* fluorescence on a blue-screen. Additionally, pTS57 contains the hygromycin B phosphotransferase (*hph*) expressed by the native *T. aurantiacus tef*-*1* promotor and *trpC* terminator. Plasmids were isolated after assembly and electroporation into MEGAX DH10B T1R Electrocomp Cells (Thermo Fisher Scientific, Waltham, MA, United States) with the QIAprep Spin Miniprep Kit (Qiagen) and transformed into *A. tumefaciens* strain EHA105 through electroporation. The plasmid pTS67 (see Table 1) was also used, which was derived from pTS57 by the above-mentioned procedure to constitutively express the transcription factor *xlnR*.

ATMT-compatible CRISPR/Cas9 plasmids were designed to target the *pyrG* gene in the target host *T. aurantiacus*. The target sequences were obtained from JGI mycocosm. All plasmid maps were designed using the software Geneious 11.1.2 (https://www.geneious.com). The gRNAs used in this study were designed using the CRISPOR algorithm [62] (http://crispor.tefor.net) to obtain predicted guide sequences for PAMs in the target gene. Three different gRNA sequences (protospacers) with no predicted off-targets were chosen and tested in vitro for correct cleavage of the target sequence by Cas9 endonuclease before performing in vivo transformation experiments [47]. All steps for the gRNA synthesis were followed according to the GeneArt Precision gRNA Synthesis Kit (Thermo Fisher Scientific, Waltham, MA, United States). Then, the in vitro Cas9 cleavage was performed using a previously amplified target *pyrG* amplicon following the steps of the Guide-it™ sgRNA In Vitro Transcription and Screening Systems User Manual (Takara Bio USA, Inc., Mountain View, CA, United States).

The two gRNAs with the highest cleavage efficiency (gRNA1 and 3) were inserted into vector pFC334 and then combined with the Cas9 gene from pFC332 via USER Cloning as described by Nødvig et al. [47]. The two *A. niger* vector backbones intended to be combined via USER Cloning (pFC332 and pFC334) were obtained from Addgene (http://www.addgene.org). Vector pFC332 carries a *cas9* gene codon optimized for expression in *A. niger* and is followed by a SV40 nuclear localization signal, which is expressed by the constitutive *tef1* promoter and *tef1* terminator. The vector also carries an *hph* gene cassette for selection as well as an AMA1 sequence responsible for replication in different fungi as described in Nødvig et al. [47]. The gRNA from vector pFC334 is expressed by two self-splicing flanking ribozymes, namely the 5′ hammer-head ribozyme HH and the 3′ Hepatitis delta virus ribozyme through the *gdpA* promoter and *trpC* terminator. USER Cloning required two PCR fragments to be fused into the pFC332 vector: The first PCR fragment contains the 20 bp protospacer; the second fragment contains a 6 bp homology to the protospacer. Complementary ends of the pFC332 for integration of the PCR fragments were obtained through restriction enzyme digestion of the vector with PacI and Nt.BbvCI (for more details see [47]). The resulting plasmids of the USER cloning procedure were named JP36_1 (gRNA1) and JP36_3 (gRNA3), which did not yet have the required elements for ATMT. Those plasmids were then used as templates to insert their gRNA–cas9 expression cassette into the ATMT vector pTS57 through Gibson Assembly. Specific primers were designed and used with NEB Q5 Hot Start High-Fidelity DNA Polymerase to amplify the gene cassettes of JP36_1 and JP36_3 from the *gpdA* promoter to the *tef1* terminator which included *cas9* and the gRNA expression cassette, as well as overhangs for the pTS57 vector. The ATMT-compatible Cas9 plasmids originating from this assembly were pJP1 (gRNA1) and pJP3 (gRNA3).

### ATMT transformation procedure

*Thermoascus aurantiacus* ascospore preparation and *A. tumefaciens* cultivation were performed as described above. The solid and liquid *A. tumefaciens* induction medium contained 200 µM acetosyringone (induction medium: salts, phosphor buffer, MES buffer, glucose, thiamine, acetosyringone and water, see [38]). All reagents were sterile filtered with Corning filter systems or small filters and a sterile syringe. The pH of the induction medium was adjusted to pH = 5.

A modified version of the ATMT procedure for *Rhodosporidium toruloides* was used [38]. Briefly, ascospores of *T. aurantiacus* were harvested from 6-day-old PDA plates and counted with a hemocytometer. *A. tumefaciens* EHA105 was grown overnight in 10 ml of liquid LB medium containing 50 µg/ml kanamycin. From this culture, a new liquid LB–kanamycin culture was generated with optical density at 600 nm (OD_600_) of 0.5 that was grown to OD_600_ = 1 and then pelleted, washed three times with induction medium, resuspended in induction medium and incubated for further 24 h. Freshly harvested fungal spores and *A. tumefaciens* cell cultivated in induction medium overnight were filtered onto a 0.45 µm cellulose acetate membrane (0.45 µm MCE Membrane, MF-Millipore) and incubated on induction medium agar plates for 2 days. The spores and cells were washed off with a wash solution containing 200 µg/ml of cefotaxime and were spread on PDA plates containing 200 µg/ml of cefotaxime and 50 µg/ml of hygromycin B with subsequent incubation for 3 days at 45 °C. Colonies were isolated and grown on a fresh PDA plates containing 200 µg/ml of cefotaxime and 50 µg/ml of hygromycin B to remove untransformed spores through harvesting ascospores from the proximate region for generating cryostocks and performing further strain tests. For Cas9 tests, those colonies were then screened for 5-FOA resistance due to CRISPR-mediated mutations in *pyrG* on Vogel’s minimal medium containing 2% sucrose, 1 mg/ml 5-FOA and 1 mg/ml uracil.

### Strain tests and screening of transformants

For cellulase and xylanase activity tests, strains isolated from hygromycin B PDA plates were used to inoculate McClendon’s medium, 0.8% SMP and a carbon source as indicated (Avicel cellulose or no carbon added). For enzyme assays, 0.8 ml of the culture broth was filtered through a spin filter column (Mini Spin Column, EconoSpin). The enzyme assays were performed on a Biomek FX through a DNS method. The first step involved manually adding 75 µl of 1% w/v Beechwood xylan (Megazyme, Bray Ireland) solution to a 96-well PCR plate (FLAT 96 WELL PCR PLATE, VWR) and 5 µl of enzyme solution. The Biomek FX was used to add DNS reagent to the PCR plates. Upon incubation of these plates at 95 °C, the plate content was transferred with Biomek FX to a flat bottom 96-well plates, and the absorbance was measured at 540 nm. d-glucose was used as a standard for the CMCase assay and D-xylose for the xylanase assay. Uracil auxotrophic strains were isolated on 5-FOA agar as described above and inoculated in PD broth containing 1 g/L uracil.

For strain verification, the mycelium DNA was extracted with the Maxwell RSC Plant DNA Kit on the Maxwell RSC Instrument (Promega, Maidson, WI, USA) according to the manual. One modification involved bead beating of intact mycelium with 300 µl extraction buffer. The concentration of the isolated DNA was measured with NanoDrop 2000 and used for PCR verifications of successful transformation. All sequencing verification was performed through Sanger sequencing.

### Sexual crossings

The mutant *T. aurantiacus* strains taRG008 (hygromycin B resistant) and FOAR2 (5-FOA resistant) were first grown individually as described above. A PDA-uracil petri dish was divided in four quarters and spore suspensions from taRG008 and FOAR2 were spotted on the middle of each quarter in an alternating fashion (Additional file 1: Fig. S1). Incubation was performed at 45 °C for six days. Once a lawn of ascospores was produced, spores were scraped off at the interface of the two crossing strains with a sterile spatula, transferred into 750 μL sterile H_2_O, vortexed, and filtered through a sterile filter tip stuffed with miracloth. A dilution was prepared and plated onto a 12 × 12 cm square plate with Vogel’s minimal medium supplemented with 1 g/L 5-FOA, 1 g/L uracil and 50 µg/mL hygromycin B in triplicates. After incubation at 45 °C for 3 days, growth was visible. Randomly picked colonies were isolated and grown on the same media as the isolation plates. Genomic DNA was extracted from isolated colonies and used for PCR-based verification purposes.

## Supplementary information

**Additional file 1.** Additional tables and figures.

## Data Availability

Plasmids (Additional file 1: Table S1) and strains (Additional file 1: Table S2) are available from the JBEI Public Registry (public-registry.jbei.org). The dataset (*T. aurantiacus* genome) supporting the conclusions of this article is available in the Mycocosm repository (https://mycocosm.jgi.doe.gov/Theau2/Theau2.home.html). All other data generated or analyzed during this study are included in this manuscript and its additional file.

## Abbreviations

ATMT: *Agrobaacterium tumefaciens*-mediated transformation
PDA: Potato dextrose agar
YPD: Yeast extract–peptone–dextrose
HDR: Homology-directed repair
