# Supplementary material for: Development of genetic tools for the thermophilic filamentous fungus Thermoascus aurantiacus
Source: Biotechnol Biofuels. 2020 Oct 10;13:167. doi: 10.1186/s13068-020-01804-x (PMC7547499; doi:10.1186/s13068-020-01804-x)
Supplement: Supplementary file 1 — Additional file 1. Additional tables and figures. [file 13068_2020_1804_MOESM1_ESM.docx]

**Development of genetic tools for thermophilic filamentous fungus *Thermoascus aurantiacus***

**Raphael Gabriel^1,2,3*^, Julia Prinz^1,2,4^, Marina Jecmenica^1,2,5,6^, Carlos Romero-Vazquez^1,2,7^, Pallas Chou^1,2,8^, Simon Harth^1,2,9^, Lena Floerl^1,2,4^, Laure Curran^1,2,10^, Anne Oostlander^1,2,3^, Linda Matz^1,2,3^, Susanne Fritsche^1,2,11^, Jennifer Gorman^1,2^, Timo Schuerg^1,2^, André Fleißner^3^ and Steven W. Singer^1,2*^**

Additional Information

Additional Tables S1-S4

Additional Figures S1-S2

**Additional Tables**

**Additional Table S1**: List of plasmids used in this study

| Name | Bacterial marker | fungal marker | Parent Plasmid | Insert | JBEI Registry |
| --- | --- | --- | --- | --- | --- |
| pTS57 | Kan | *hph* | NA | *gfp* | JPUB_017131 |
| pTS67 | Kan | *hph* | pTS57 | *xlnR* | JPUB_017129 |
| pJP1 | Kan | *hph* | pTS57, JP36_1 | *cas9, gRNA 1* | JPUB_017147 |
| pJP3 | Kan | *hph* | pTS57, JP36_3 | *cas9, gRNA 3* | JPUB_017149 |

**Additional Table S2**: List of strains derived from *T. aurantiacus* ATCC 26904 used in this study

| Name | Genotype | Phenotype | JBEI Registry ID |
| --- | --- | --- | --- |
| hph-1 | *hph* ectopic integration | Resistance to  hygromycin B | JPUB_017144 |
| hph-2 | *hph* ectopic integration | Resistance to  hygromycin B | JPUB_017145 |
| taRG003 | *xlnR* ectopic integration | Xylanase hyperproduction | JPUB_017132 |
| taRG005 | *xlnR* ectopic integration | Xylanase hyperproduction | JPUB_017133 |
| taRG010 | *xlnR* ectopic integration | Xylanase hyperproduction | JPUB_017134 |
| taRG015 | *xlnR* ectopic integration | Xylanase hyperproduction | JPUB_017135 |
| taRG020 | *xlnR* ectopic integration | Xylanase hyperproduction | JPUB_017136 |
| taRG023 | *xlnR* ectopic integration | Xylanase hyperproduction | JPUB_017137 |
| taRG025 | *xlnR* ectopic integration | Xylanase hyperproduction | JPUB_017138 |
| taRG027 | *xlnR* ectopic integration | Xylanase hyperproduction | JPUB_017139 |
| taRG028 | *xlnR* ectopic integration | Xylanase hyperproduction | JPUB_017140 |
| taRG029 | *xlnR* ectopic integration | Xylanase hyperproduction | JPUB_017141 |
| FOAR1 | UV-induced *pyrE* insertion | 5-FOA resistant, uracil auxotroph | JPUB_017142 |
| FOAR2 | UV-induced *yre* SNP | 5-FOA resistant, uracil auxotroph | JPUB_017143 |
| FOAR2 *x* taRG008 | Sexual cross of *yre* SNP and *hph* insertion | 5-FOA resistant, uracil auxotroph, hygromycin B resistant | JPUB_017156 |
| JP1-1 | Cas9-induced pyrG mutation | 5-FOA resistant, uracil auxotroph | JPUB_017150 |
| JP1-2 | Cas9-induced pyrG mutation | 5-FOA resistant, uracil auxotroph | JPUB_017151 |
| JP1-3 | Cas9-induced pyrG mutation | 5-FOA resistant, uracil auxotroph | JPUB_017152 |
| JP1-4 | Cas9-induced pyrG mutation | 5-FOA resistant, uracil auxotroph | JPUB_017153 |
| JP1-5 | Cas9-induced pyrG mutation | 5-FOA resistant, uracil auxotroph | JPUB_017154 |
| JP1-6 | Cas9-induced pyrG mutation | 5-FOA resistant, uracil auxotroph | JPUB_017155 |

**Additional Table S3**: List of primers used in this study

| PCR | | name | Sequence |
| --- | --- | --- | --- |
| *hph1* | FWD | RG1 | CTCGGAGGGCGAAGAATCTC |
|  | REV | RG2 | ATTTGTGTACGCCCGACAGT |
| *hph2* | FWD | TS222 | CGTAGTACCTGAGCACCCCTCTGAGCTCTT |
|  | REV | TS223 | CCATTTGTCTCAACTCCGGAGCTGACATCGA |
| *pyrE* | FWD | RG75 | GACGGTTTCTATACAGTCTTTTCAG |
|  | REV | RG76 | CCCCCGATGTTACTCCGC |
| *pyrG* | FWD | LLK683 | TTCTTACTACAACTTGGCAACCTTC |
|  | REV | LLK686 | ACAAGCCAAATTACCAGCAGAATAC |

**Additional Table S4**. List of protospacers and PAM sequences used in this study

| **Target locus** | **ID** | **Protospacer sequence (5'-3')** | **PAM (5'-3')** |
| --- | --- | --- | --- |
| *pyrG* | gRNA 1 | CTTTTGCGCGCGAGCGCCGT | AGG |
| *pyrG* | gRNA 2 | GAGTCTTCCTGCACAGGCCT | GGG |
| *pyrG* | gRNA 3 | TCGGCGCCCGACTTCCCCTA | CGG |

**Additional Figures**

**Additional Figure S1**


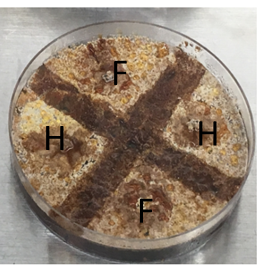


**Additional Fig. S1:** Outcrossing of *T. aurantiacus*. Image of the plate setup for strain crossings: the two parent strains were plated in alternating fashion (F: 5-FOA resistant parent strain FOAR2, and H: hygromycin B resistant parent strain taRG008).

**Additional Figure S2a,b**


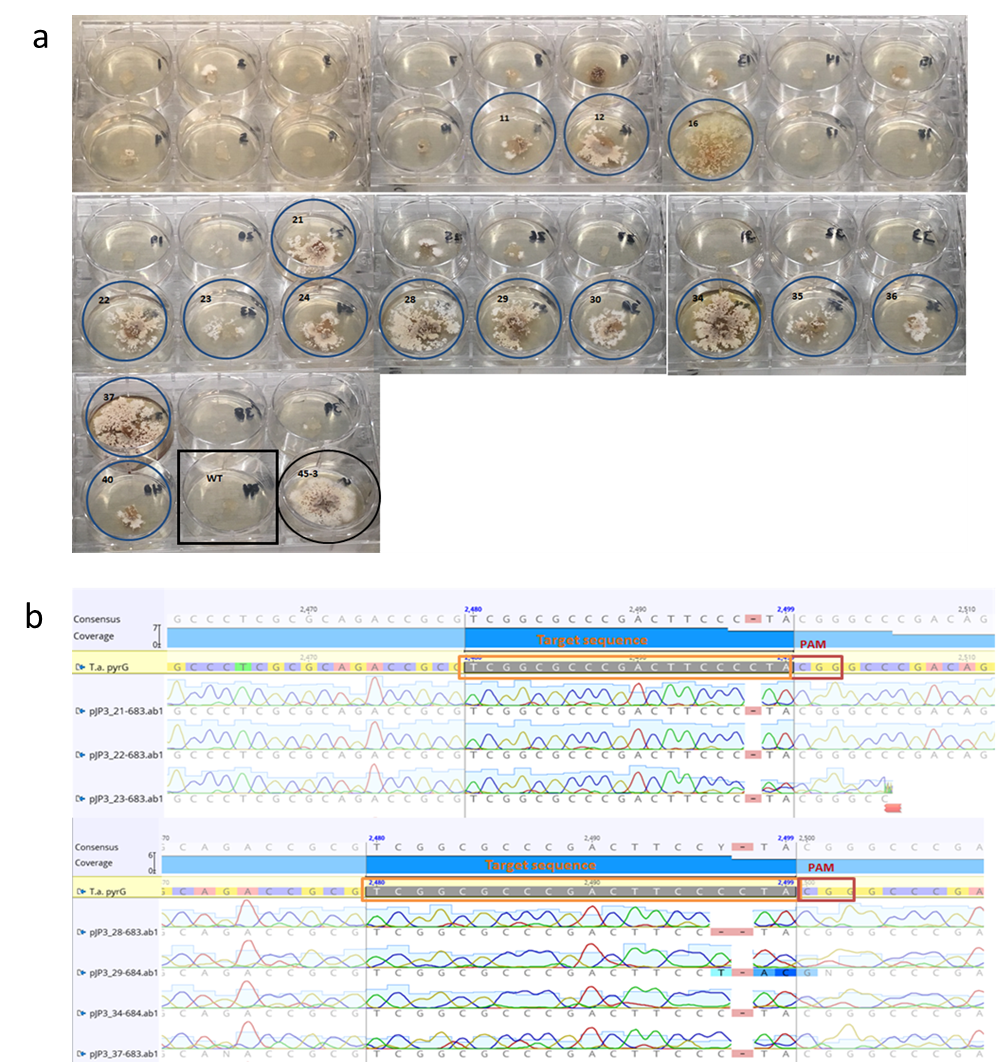


**Additional Fig. S2:** Screening for *pyrG* deletion strains on 5-FOA uracil medium. (a) A subset of 20 colonies from ATMT transformations using pJP1 (gRNA 1) and pJP3 (gRNA 3) were selected for 5-FOA resistance each. The wild type (black square frame) and FOAR2 as a 5-FOA resistant positive control (black round frame) were included. pJP1 colony 11, 12 and 16 as well as pJP3 colony 21, 22-24, 28-30, 34-37 and 40 were positive transformants on the selection medium and were used for Sanger sequencing verification procedures. (b) Sanger sequencing results for *T. aurantiacus* pJP3 transformants revealing deletions and mismatches through Cas9 cleavage next to the PAM sequence (framed in red) in the *pyrG* target sequence (framed in orange). The sequence analysis was performed with Geneious version 11.1 (Biomatters).
